# Toxoplasmosis-associated IRIS involving the CNS: a case report with longitudinal analysis of T cell subsets

**DOI:** 10.1186/s12879-016-2159-x

**Published:** 2017-01-13

**Authors:** Rita Rb-Silva, Claudia Nobrega, Eugénia Reiriz, Soraia Almeida, Rui Sarmento-Castro, Margarida Correia-Neves, Ana Horta

**Affiliations:** 1 Life and Health Sciences Research Institute (ICVS), School of Medicine, University of Minho, Campus de Gualtar, 4710-057 Braga, Portugal; 2 ICVS/3B’s, PT Government Associate Laboratory, Braga/Guimarães, Portugal; 3 Department of Infectious Diseases of Centro Hospitalar do Porto, Hospital Joaquim Urbano Unit, Porto, Portugal

**Keywords:** Human immunodeficiency virus, Immune reconstitution inflammatory syndrome, Toxoplasmosis, T cell subsets, Regulatory T cells

## Abstract

**Background:**

HIV-infected patients may present an unforeseen clinical worsening after initiating antiretroviral therapy known as immune reconstitution inflammatory syndrome (IRIS). This syndrome is characterized by a heightened inflammatory response toward infectious or non-infectious triggers, and it may affect different organs. Diagnosis of IRIS involving the central nervous system (CNS-IRIS) is challenging due to heterogeneous manifestations, absence of biomarkers to identify this condition, risk of long-term sequelae and high mortality. Hence, a deeper knowledge of CNS-IRIS pathogenesis is needed.

**Case presentation:**

A 37-year-old man was diagnosed with AIDS and cerebral toxoplasmosis. Anti-toxoplasma treatment was initiated immediately, followed by active antiretroviral therapy (HAART) 1 month later. At 2 months of HAART, he presented with progressive hyposensitivity of the right lower limb associated with brain and dorsal spinal cord lesions, compatible with paradoxical toxoplasmosis-associated CNS-IRIS, a condition with very few reported cases. A stereotactic biopsy was planned but was postponed based on its inherent risks. Patient showed clinical improvement with no requirement of corticosteroid therapy. Routine laboratorial analysis was complemented with longitudinal evaluation of blood T cell subsets at 0, 1, 2, 3 and 6 months upon HAART initiation. A control group composed by 9 HIV-infected patients from the same hospital but with no IRIS was analysed for comparison. The CNS-IRIS patient showed lower percentage of memory CD4^+^ T cells and higher percentage of activated CD4^+^ T cells at HAART initiation. The percentage of memory CD4^+^ T cells drastically increased at 1 month after HAART initiation and became higher in comparison to the control group until clinical recovery onset; the percentage of memory CD8^+^ T cells was consistently lower throughout follow-up. Interestingly, the percentage of regulatory T cells (Treg) on the CNS-IRIS patient reached a minimum around 1 month before symptoms onset.

**Conclusion:**

Although both stereotactic biopsies and steroid therapy might be of use in CNS-IRIS cases and should be considered for these patients, they might be unnecessary to achieve clinical improvement as shown in this case. Immunological characterization of more CNS-IRIS cases is essential to shed some light on the pathogenesis of this condition.

**Electronic supplementary material:**

The online version of this article (doi:10.1186/s12879-016-2159-x) contains supplementary material, which is available to authorized users.

## Background

Immune reconstitution inflammatory syndrome (IRIS) occurs in a substantial proportion of patients with human immunodeficiency virus (HIV) infection during the first months of highly active antiretroviral therapy (HAART) [1]. IRIS can be associated to a concomitant autoimmune disorder, an immune-mediated inflammatory condition or, most frequently, an infectious disease. In the last case, IRIS is classified as unmasking or paradoxical, depending on the infection being subclinical/undiagnosed or previously diagnosed and treated, respectively [1, 2]. IRIS incidence varies depending on the underlying infection, reaching up to 40% in HIV-infected patients with cytomegalovirus retinitis [3]. Low CD4^+^ T cell count at HAART onset and high load of an opportunistic pathogen are the most commonly identified risk factors for IRIS [4]. Although most cases are self-limited, long-term sequelae and fatal outcomes may occur, particularly when neurologic structures are involved [5].

The immunopathogenesis underlying infection-associated IRIS is seldom understood and seems to depend on the underlying pathogen. Although IRIS studies suggested an involvement of monocytes and NK cells [6, 7], cells of the adaptive immune system have been considered the major players. There is evidence that expansion of pathogen-specific CD4^+^ T cells accompanies most IRIS events [8], but differences on T cell responses have not been consistently identified between patients with or without IRIS [9]. In addition, quantitative and qualitative defects on regulatory T cells (Treg), a specific subset of CD4^+^ T cells known for its immune suppressive effect, have been associated with IRIS [10]. Most studies regarding IRIS immunopathogenesis are based on tuberculosis-associated IRIS cases, so reports of other types of IRIS to explore the underlying responsible mechanisms are of great interest.

Encephalitis caused by *Toxoplasma gondii* is one of the most common life-threatening central nervous system (CNS) infections in patients with acquired immunodeficiency syndrome (AIDS) [11]. Infection by *T. gondii* is characterized by an asymptomatic acute phase that may be followed by the dissemination of cysts, mainly to muscles and brain. CNS toxoplasmosis most often results from reactivation of the infection, probably due to the severely depressed T cell-mediated immune response and imbalanced interactions between intracerebral T cells, recruited myeloid cells and brain-resident cells, as suggested by mouse models [12, 13]. CD4^+^ and CD8^+^ T cells have been described as the main players in the host’s resistance to this infection [14].

Despite the significant incidence of cerebral toxoplasmosis, only five paradoxical CNS-IRIS cases associated to *T. gondii* have been previously described (Table 1) [15–18]. Similarly to other IRIS conditions, there is no consensual treatment for toxoplasmosis-associated IRIS and prognosis is poor [5]. For these reasons, a better understanding of the immunopathology is needed to find biomarkers for early detection and to help developing targeted therapies leading to a consequent prognosis improvement. We report here the sixth case of paradoxical toxoplasmosis-associated CNS-IRIS and describe for the first time the evolution of different T cell subsets in the peripheral blood of the patient.

**Table 1:** Review of the reported clinical cases of paradoxical CNS-IRIS associated to toxoplasmosis^a^

	Toxoplasmosis	CNS-IRIS	Ref.
Case 1 Female, 30 yo	**At diagnosis:** HIV infection for 6 months, not on HAART. **Manifestations:** Fever, left hemiparesis with the Babinski sign. **Brain MRI:** Ring-enhancing mass in the right basal nuclei. **Treatment:** ATT with trimethoprim–sulfamethoxazole, corticosteroids, HAART. **Response to treatment:** Clinical and radiographic improvement.	**Presentation:** Approximately 6 weeks after toxoplasmosis diagnosis, patient was readmitted with headaches (for 2 weeks), imbalance, and left hemiparesis (for 48 h). **Brain MRI:** Mass persistence, more vasogenic edema and new, bilateral but smaller contrast enhancing lesions. **Brain biopsy:** Abundant tachyzoites. **Treatment:** Reduction of the corticosteroids tapering rate. **Outcome:** Clinical improvement without complete resolution (2 months after IRIS diagnosis).	[15]
Case 2 Female, 26 yo	**At diagnosis:** HIV infection for 8 years, not on HAART. History of cerebral toxoplasmosis 4 years before. **Manifestations:** Ataxia, left-sided weakness and hyperreflexia for 1 month. **Brain CT scan:** Scattered calcified lesions with no perilesional edema or contrast enhancement. **CSF analysis:** Negative PCR for *T. gondii*. **Brain MRI:** Multiple areas of high signal intensity on fluid-attenuated inversion recovery (FLAIR) images, some presenting nodular or ring enhancement. **Treatment:** ATT and HAART.	**Presentation:** After a steady clinical period of 1 month, progression of symptoms. **Brain MRI:** Enlargement of most of the lesions, mainly with perilesional high signal intensity on FLAIR images, as well as stronger contrast enhancement. **Brain biopsy:** Collections of histiocytic giant multinucleated cells. Marked perivascular lymphocytic infiltrates with a predominance of CD8^+^ T cells. Reactive gliosis. No *T.gondii* cysts or tachyzoites. **Treatment:** Maintenance of therapeutic measures. No corticotherapy. **Outcome:** Clinical improvement.	[16]
Case 3 Male, 34 yo	**At diagnosis:** HIV infection, non-compliant with HAART. **Manifestations:** Lower extremities weakness for 6 months, sensory level at L4 and constipation. **Toxoplasma serum IgG level:** Increased. **CSF studies:** Negative.^b^ **Brain and spine MRI:** No contrast-enhanced brain lesions. Expansive intramedullary enhancing lesion in spine, at T11 through T12. **Treatment:** Laminectomy and surgical spinal cord decompression, corticosteroids, ATT, HAART. **Pathology of the excised spinal lesion:** *T. gondii* cysts. **Response to treatment:** Clinical improvement.	**Presentation:** Worsening of weakness 3 weeks after treatment initiation. Cachexy, dysarthria, hypotension and areflexia in upper and lower extremities after one month approximately. **Brain and spine MRI:** Two new enhancing lesions in brain. No new lesions in spine. **CSF studies:** All within normal. **Electromyogram/nerve conduction study:** Results consistent with a sensorimotor neuropathy superimposed on a predominantly proximal myopathic process. **Muscle biopsy:** Necrosis, lymphocytic and plasma cell infiltrates with abundant *T. gondii* cysts.^c^ **Outcome:** Multiorgan dysfunction and death 2 weeks after the diagnosis of toxoplasmosis myositis.	[17]
Case 4 Male, 35 yo	**At diagnosis:** AIDS previously diagnosed, not on HAART or prophylaxis. **Manifestations:** Left upper extremity weakness for 3 weeks, associated to fever and respiratory symptoms for 1 week (concomitant respiratory infection). **Brain MRI:** Two ring-enhancing lesions in the right precentral and occipital temporal areas. **Treatment:** Ceftriaxone and azithromycin, ATT, HAART.	**Presentation:** Progression of upper extremity weakness during the first 2 weeks on HAART. **CSF studies:** 6 WBC/mm^3^ (96% lymphocytes; 4% monocytes); positive EBV PCR. **Brain MRI:** Enlargement of the two prior lesions and development of a third lesion. **Brain biopsy:** Rare *T. gondii* tachyzoites and numerous bradyzoites. CD8^+^ predominant lymphocytic infiltrates. **Treatment:** Corticosteroids. Outcome: Clinical improvement without complete resolution.	[18]
Case 5 Male, 51 yo	**At diagnosis:** AIDS previously diagnosed, not on HAART or prophylaxis. **Manifestations:** Unsteady gait, left upper extremity weakness, headaches, weight loss and fever for 2 weeks. **Brain MRI:** Multiple ring-enhancing lesions in his fronto parietal region. **Treatment:** ATT, HAART.	**Presentation:** After an initial improvement, there was progression of neurological symptoms around 2 weeks after treatment initiation. **CSF studies:** 6 WBC/mm^3^ (100% lymphocytes); no malignant cells. **Brain MRI:** No significant change. **Brain biopsy:** Presence of *T. gondii.* CD8^+^ predominant lymphocytic infiltrates. **Treatment:** Corticosteroids. **Outcome:** Clinical improvement.	[18]

Cases are ordered by year of publication. ^a^All case descriptions reported infection by *Toxoplasma gondii*, except for case 2 (no species was specified). ^b^No specification for *T. gondii.* ^c^IRIS treatment not available

*AIDS* acquired immunodeficiency syndrome, *ATT* anti-toxoplasma therapy (unless otherwise stated, with sulfadiazine, pyrimethamine and folic acid), *CNS-IRIS* central nervous system immune reconstitution inflammatory syndrome, *CSF* cerebrospinal fluid, *CT* computed tomography, *EBV* Epstein-Barr virus, *HAART* highly active antiretroviral therapy, *HIV* human immunodeficiency virus, *MRI* magnetic resonance imaging, *PCR* polymerase chain reaction, *T. gondii Toxoplasma gondii*, *WBC* white blood cells, *yo* year-old

## Methods

### Patients

In addition to the *T. gondii* CNS-IRIS clinical case, a control group was selected (Table 2) based on the following inclusion criteria: 1) baseline CD4^+^ T cell count <100/μL; 2) absence of AIDS-defining conditions at baseline; 3) absence of IRIS. Participants were all over 18 years old; chronically infected with HIV-1 (referred as HIV from now on) and enrolled in the study at the moment of HAART initiation. The time-points considered for the present analysis were: 0 (or baseline), 1, 2, 3 and 6 months after HAART initiation. HAART schemes chosen for each individual (Table 2) took into consideration: scientific policy; national and international guidelines [19]; characteristics of each individual; and drug cost. Information regarding ethical considerations are available in the “Ethics approval and consent to participate” section at the end of this report.

**Table 2:** Demographic and clinical characteristics of the patients at baseline

	IRIS case	Control Group (*n* = 9)
**Gender**, Male, % (n)	Male	67% (6)
**Age at baseline** in years, Median [min; max]	37	44 [28; 48]
**HIV transmission mode**, % (n)		
Intravenous drug user		33% (3)
Men who have sex with men (MSM)	MSM	22% (2)
Heterosexual		44% (4)
**Log**_**10**_ **of HIV viral load at baseline** in copies per mL, Median [min; max]	5.5	5.4 [4.9; 6.4]
**CD4** ^**+**^ **/μL at baseline**, Median [min; max]	20	25 [8; 97]
**HAART regimen components**		Patient 1: (TDF + FTC) + DRVr Patients 2–7: (TDF + FTC) + EFV Patients 8–9: (ABC + 3TC) + EFV
2 nucleoside or nucleotide analogue reverse transcriptase inhibitors + 3^rd^ drug	(TDF + FTC) + DRVr	

*ABC* abacavir, *DRVr* ritonavir boosted darunavir, *EFV* efavirenz, *FTC* emtricitabine, *HAART* Highly active antiretroviral therapy, *HIV* human immunodeficiency virus, *TDF* tenofovir disoproxil fumarate, *3TC* lamivudine

### Flow cytometry

Venous blood samples were drawn into Na_2_-EDTA collecting tubes and processed on the same day. The evaluation of T cell subsets (except Treg) was performed in 200 μL of whole blood upon 15 min incubation with a combination of antibodies specific for CD3 (clone OKT3), CD4 (clone RPA-T4), CD8 (RPA-T8), CD45RA (clone HI100), CD45RO (clone UCHL1), CD69 (clone FN50) and HLA-DR (clone L243; all from BioLegend, San Diego, CA, USA). Erythrocytes were lysed upon incubation with Lysis Buffer (BD Biosciences, San Jose, CA, USA) for 15 min and cells were washed. Treg analysis was performed in peripheral blood mononuclear cells (PBMCs) isolated from whole blood by gradient centrifugation. Two million PBMCs were stained with antibodies specific for CD3 (clone UCHT1), CD4, CD25 (clone BC96), CD31 (clone WM59) and CD127 (clone AO19D5; all from BioLegend). Afterwards, cells were washed, fixed, permeabilized and stained for the intracellular markers FOXP3 (clone PCH101; eBioscience, San Diego, CA, USA) and Ki67 (clone MOPC-21; BD Biosciences), as described elsewhere [20]. Samples were acquired on a BD™ LSRII Flow Cytometry System using BD FACSDiva software (Franklin Lakes, NJ, USA). Data were analysed using FlowJo software V.10 (Ashland, OR, USA) accordingly to the gating strategies represented in Additional file 1: Figures S1 and S2.

### Statistical analysis

Assessment of the distribution of the different variables was performed by the Shapiro-Wilk Normality Test. Comparisons between the control group and the clinical case were performed by One Sample *t*-Test or by Wilcoxon Signed Rank Test, depending on the normal or not normal distribution of the variables in the control group, respectively. Statistical analyses were performed using the IBM SPSS v.22 software (Armonk, NY, USA). Differences were considered statistically significant when *p* < 0.05 and graphically marked with * when 0.05 > *p* > 0.01 or ** when *p* ≤ 0.01. Detailed information on the statistical analysis is supplied in the Additional file 1: Tables S1 and S2.

## Case presentation

**Fig. 1:**
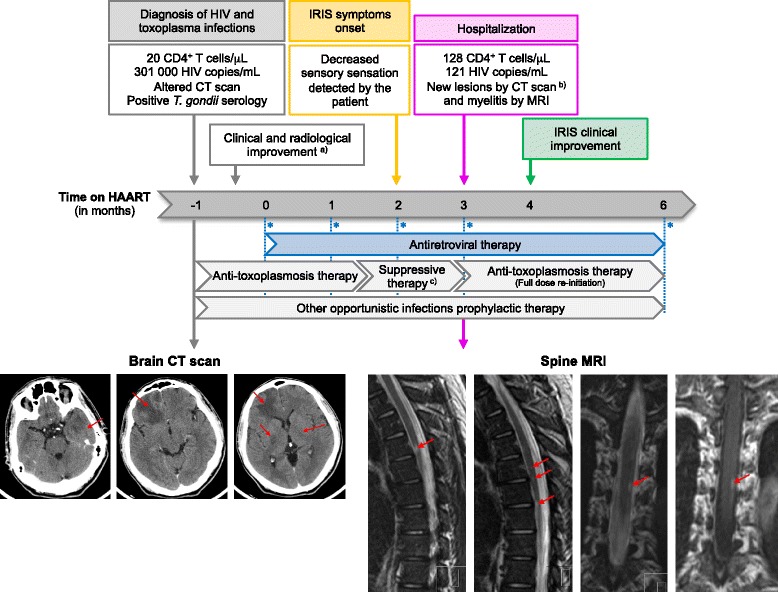
CNS-IRIS case timeline. On top, a timeline of the clinical evolution shows the temporal relation between toxoplasmosis CNS-IRIS symptoms onset (*yellow arrow*); the routine medical appointment followed by hospital admission (*pink arrow*) and the beginning of the clinical recovery (*green arrow*). *Blue* asterisks mark the five time-points when complete clinical and laboratorial evaluation was performed and blood samples were collected for flow cytometry analysis. On bottom left, brain CT performed at the initial diagnosis of toxoplamosis. On bottom right, spinal cord MRI performed after hospital admission. ^a, b)^ See Additional file 1: Figure S3 and S4, respectively. ^c)^ Suppressive therapy: long-term, low-dose anti-toxoplasma therapy to prevent further recurrent episodes

A 37-year-old man was diagnosed with HIV infection and cerebral toxoplasmosis. The patient presented headache, psychomotor retardation and left hemiparesis, associated to advanced immunodeficiency (20 CD4^+^ T cells/μL), high plasma HIV load (301,000 copies/mL) and positive serology for toxoplasmosis. A computed tomography (CT) scan showed scattered lesions in the brain parenchyma with perilesional edema, sulcal effacement suggesting mass effect, and some ring contrast enhancement (Fig. 1). After initiation of specific anti-toxoplasma treatment, without corticosteroid therapy, a clinical and radiologic improvement was observed (Additional file 1: Figure S3). HAART was started 1 month after HIV diagnosis (drug combination on Table 2) and toxoplasmosis treatment was maintained for further 1.5 months and then changed to suppressive therapy (long-term, low-dose therapy to prevent further recurrent episodes); primary prophylaxis for other opportunistic infections was maintained (Fig. 1).

In a routine medical appointment 3 months after HAART initiation, the patient presented decreased sensation of the right lower limb that started about 1 month before. The hyposensitivity started abruptly, with progressive worsening, extending at that moment to the entire right lower limb and half of the abdomen. The patient showed neither fever, nor symptoms of the respiratory, digestive or urinary systems. He stated being compliant to all prescribed therapies (*i.e.,* HAART, therapy for toxoplasmosis and primary prophylaxis for *Mycobacterium avium* complex disease). Neurological examination, mental status and cranial nerves exam were normal. No papilledema or neck stiffness was present. Besides a slight sequelar left hemiparesis, an upper right unilateral sensory level at D-9 was present with loss of touch sensation and of the ability to feel pain below this level across the entire right lower limb and right hemi-abdomen. Proprioception, muscular force and reflexes were present in the correspondent area. The patient was immediately admitted to the hospital. A brain CT scan was performed and showed the previous lesions (a couple revealing worsening with a slight ring contrast enhancement and perilesional edema, others revealing improvement) and some new lesions (Additional file 1: Figure S4). A magnetic resonance imaging (MRI) of the dorsal spinal cord revealed myelitis: multiples areas of high signal intensity on the T2-weighted images, located between D3 and D9, and probably a posterolateral lesion at D7-D8 (Fig. 1). At this moment (*i.e.,* 3 months after HAART initiation), the effect of therapy on the CD4^+^ T cells and viral load was already evident; CD4^+^ T cell count was 128 cells/μL and the plasma viral load of 121 copies/mL. The cerebrospinal fluid (CSF) evaluation showed: 5 cells/μL; 58 mg/dL protein; 51 mg/dL glucose; negative results for Gram and acid-fast stains, microbiological cultures, cryptococcal antigen, Venereal Disease Research Laboratory (VDRL) test and polymerase chain reactions (PCR) to identify other common agents (JC virus, BK virus, cytomegalovirus, herpes simplex virus, human herpesviruses 6, varicella-zoster virus, and enterovirus). A CSF PCR for *T. gondii* was not performed due to technical limitations. The CSF PCR for Epstein-Barr virus was positive, but a normal CSF lymphocyte phenotype assay and a whole-body positron emission tomography (PET) scan revealed neither hyper-metabolic cerebral nor medullar lesions, which ruled out CNS lymphoma. Serological HTLV-I/II antibody assay was negative.

The clinical and laboratory information suggested a potential case of CNS-IRIS. The differential diagnosis between IRIS and a progression of toxoplasmosis infection is difficult, but if the last hypothesis was true, most probably a marked worsening of all the previous lesions would be present in the brain CT scan. Furthermore, the patient stated that he had correctly took his anti-toxoplasma medication.

Because there are no consensual recommendations for the treatment of toxoplasmosis-associated CNS-IRIS, anti-toxoplasma therapy with full doses was restarted by precaution, HAART and prophylaxis for opportunistic infections were maintained (Fig. 1) and the patient was kept under close surveillance. Corticosteroids were not administered.

One month after hospitalization, the clinical status of the patient was stable and the results of a new brain and medullar MRI were similar to the previous one. A stereotactic brain/medulla biopsy was planned, however it was readily postpone due to its inherent technical risks as soon as the patient revealed signals of clinical improvement. A MRI was repeated 4 months later showing a significant improvement of the brain lesions and a complete resolution of the medullar lesions. The patient presented fully sensory recovery about 9 months after HAART onset, only maintaining the sequelar left hemiparesis. At that time-point, CD4^+^ T cell count was 244 cells/μL and the HIV viral load was <20 copies/mL. The patient maintained in addition to HAART suppressive treatment for toxoplasmosis for 12 months.

### Longitudinal analysis of T cell subsets by flow cytometry

A detailed analysis of T cell subsets evolution was performed in the present CNS-IRIS case. Taking into consideration that this investigation encompasses a single case of a rare condition, the analysis was complemented with a group of HIV-infected patients that did not develop IRIS and were followed in the same hospital, all with <100 CD4^+^ T cells/μL at baseline and a rapid decline of the HIV plasma viral load after HAART initiation, similar to what was observed in the reported case.

**Fig. 2:**
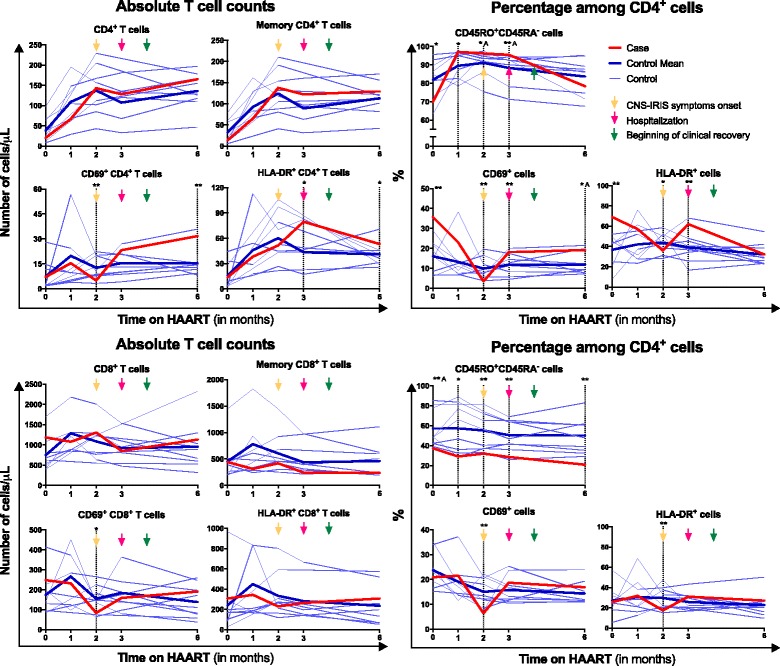
Evolution of CD4^+^ and CD8^+^ T cells and their memory and activated subsets throughout HAART. Absolute numbers of CD4^+^ T and CD8^+^ cells and their subsets of memory (CD45RA^−^CD45RO^+^) and activated (CD69^+^ or HLA-DR^+^) cells are represented on the *left graphs*, and percentages, on the *right graphs*. Gating strategy used to define these populations are depicted in Additional file 1: Figure S1. All comparisons were performed using One Sample *t*-Test, except the ones marked with ^A^, in which One-Sample Wilcoxon Signed Rank Test was used. * represents 0.05 > *p* > 0.01 and **, *p* ≤ 0.01
- T cell activation:
  At baseline, there were no differences on the absolute number of CD4^+^ or CD8^+^ T cells expressing CD69 or HLA-DR between the CNS-IRIS case and the controls. However, there was a higher percentage of both CD69^+^ cells and HLA-DR^+^ cells among CD4^+^ T cells, but not among CD8^+^ T cells, in the CNS-IRIS case. The absolute number of HLA-DR^+^ CD4^+^ T cells in the CNS-IRIS case increased from baseline (when it was similar to the controls) up to 3 months on HAART (when IRIS was diagnosed). In contrast, the percentage of HLA-DR^+^ cells among CD4^+^ T cells decreased from baseline until 2 months on HAART (at IRIS symptoms onset). After 6 months on HAART, the absolute number of CD4^+^ T cells expressing CD69 or HLA-DR was higher in the CNS-IRIS compared to the controls. This difference was not observed in CD8^+^ T cells (Fig. 2).
- Regulatory T cells:
  There was no statistically significant difference on Treg percentage among CD4^+^ T cells or absolute number between controls and the clinical case at baseline (Fig. 3). However, while the mean Treg percentage continuously decreased after HAART initiation in the controls, the CNS-IRIS patient presented first a decrease, reaching a minimum at 1 month of HAART, and then an increase of this percentage, until 3 months of HAART (when IRIS was diagnosed, the patient was hospitalized and the therapy changed). After 6 months on HAART, Treg percentage was similar between the CNS-IRIS case and the controls. Regarding Treg subsets, there was a higher absolute number and percentage of naïve Treg and recent thymic emigrants Treg in the CNS-IRIS patient, compared to the controls, at baseline and 3 months after HAART initiation.

- Naïve and memory T cells:
  No differences were observed on the absolute numbers of memory CD4^+^ or CD8^+^ T cells (CD45RA^−^CD45RO^+^) between the CNS-IRIS case and controls at any time-point. The case had a significant lower percentage of memory CD4^+^ T cells at baseline, but this difference was inverted one month later (Fig. 2). In both CNS-IRIS case and controls, there was an increase in the percentage of memory CD4^+^ T cells from baseline to 1 month, but this increase was higher in the CNS-IRIS case (1.4 fold-change) compared to the controls (1.1 mean fold-change). A higher percentage of memory CD4^+^ T cells in the CNS-IRIS patient was still present at symptoms onset and IRIS diagnosis (*i.e.* 2 and 3 months after HAART initiation).

**Fig. 3:**
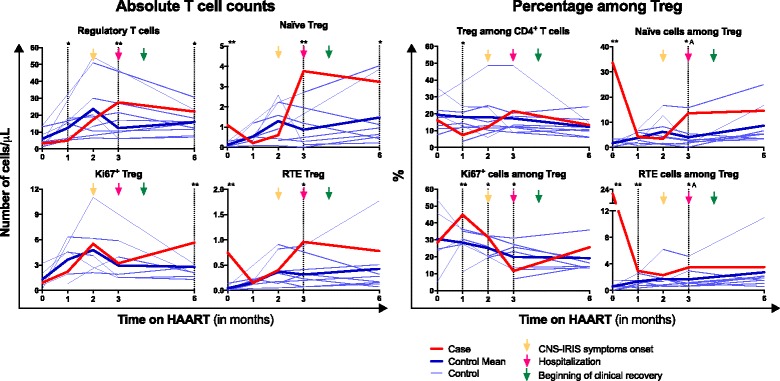
Evolution of regulatory T cells (Treg) throughout HAART. Absolute numbers of total Treg (FOXP3^+^CD25^+^CD127^−^CD4^+^ T cells), naïve (CD45RA^+^) Treg, recent thymic emigrants (RTE; CD45RA^+^CD31^High^) Treg and Treg undergoing proliferation (Ki67^+^) are represented on the *left graphs*, and percentage, on the *right graphs*. Gating strategy used to define these populations is depicted in Additional file 1: Figure S2. All comparisons were performed using One Sample *t*-Test, except the ones marked with ^A^, in which One-Sample Wilcoxon Signed Rank Test was used. * represents 0.05 > *p* > 0.01 and **, *p* ≤ 0.01
  The CNS-IRIS case presented a higher Treg proliferation rate between 1 and 2 months on HAART, compared to the controls. This difference inverted at 3 months on HAART, and was not present 3 months after (Fig. 3).

## Discussion

CNS-IRIS stands of high clinical relevance mainly because it contributes to the bulk of IRIS mortality [21] and its prevalence is most probably underestimated since diagnosis is hampered by the heterogeneous manifestations, CNS poor access to clinical investigation, absence of specific biomarkers and the need to exclude other conditions. CNS-IRIS diagnosis is presently based on a multi-parametrical assessment, including: 1) high pathogen load; 2) positive response to HAART with evidence of controlled HIV replication; 3) short temporal gap between HAART onset and disease worsening (usually up to 3 months); 4) inflammatory reaction associated with the worsening of previous lesions or the appearance of new lesions; 5) exclusion of differential diagnosis and; 6) clinical and radiological improvement without specific empiric therapies. All these criteria were observed in the case described herein. To better understand the clinical features of paradoxical toxoplasmosis-associated CNS-IRIS, we summarized the information of the previously reported 5 cases [15–18] (Table 1) and compared with the new reported case.

Contrary to the other 5 cases in which HIV infection was diagnosed before cerebral toxoplasmosis, in the present CNS-IRIS patient both conditions were diagnosed simultaneously. No differences in the immune pathogenesis or clinical evolution are expected due to this, since none was under HAART at the moment of toxoplasmosis diagnosis, except one patient that was non-compliant to therapy [17]. The neurologic symptoms of toxoplasmosis were relatively similar between the 6 cases: all patients presented weakness of upper or lower extremities. Anti-toxoplasma therapy was composed by sulfadiazine, pyrimethamine and folic acid, except in the case described by Tremont-Lukast et al*.* [15], in which the patient was treated with trimethoprim–sulfamethoxazole.

Regarding the onset of IRIS symptoms in the previously described reports, it occurred in the first 3 weeks on HAART, while in the case described herein, IRIS symptoms only appeared 2 months after HAART initiation. Still, this period is quite variable from case to case, typically from a few days to 6 months upon HAART initiation [1]. All patients performed repeated MRI, and all but one (our case) had histological studies (brain, in 4 cases; spinal medulla and muscle, in the other). Stereotactic biopsies are very important for diagnosis confirmation and treatment adjustment in HIV-infected patients [22], but there is a risk of severe complications, such as intracranial haemorrhage. The use of MRI imaging in association with a good clinical judgement and close monitoring avoided an unnecessary and perilous brain or medullar biopsy in the case here reported.

CSF evaluation was performed after IRIS symptoms onset in the present CNS-IRIS case and in 3 of the previously reported cases [17, 18]. Though these analyses focus on increased levels of anti-toxoplasma IgG or IgM or the presence of *T. gondii* DNA, the negativity of these results does not exclude toxoplasmosis, given the limited sensitivity of the techniques used.

Therapeutic strategies for IRIS are still controversial [23], and there is no controlled studies supporting the use of pharmacologic interventions in CNS-IRIS. Corticotherapy should be carefully considered in HIV-infected patients with CNS-IRIS since it increases the risk of opportunistic infections and may worsen an undiagnosed condition. Four of the 5 previously reported patients received corticosteroids: although 3 of these had a favourable evolution [15, 18], the other patient presented a fatal outcome [17]. There was no administration of corticotherapy in 2 cases (here and the one from Cabral et al. [16]) and still patients had a favourable outcome.

Case reports represent an important study design in advancing medical scientific knowledge on rare pathological conditions. While CNS-IRIS immunopathogenesis is still under active investigation, it seems to mostly depend on the T cell-mediated response [24, 25]. Therefore, we performed a longitudinal analysis of T cell subsets in the CNS-IRIS case and compared the results with the ones from a control group of HIV-infected patients. The differences need to be addressed cautiously for two main reasons: 1) only one clinical case was studied due to the fact that this condition is largely unexpected and rare; 2) the selection criteria of the control group did not exclude some underlying conditions that might impact on the assessed parameters. An interesting control group would be toxoplasma/HIV co-infected individuals who did not develop IRIS, but no such cases occurred in our cohort study, in which 85 HIV-infected patients were followed for 24 months. Notwithstanding, this represents a valuable characterization of the clinical case that might be relevant to better understand toxoplasmosis associated CNS-IRIS immunopathogenesis.

Differences already present before HAART introduction in HIV-infected patients that develop IRIS, comparing to those patients that do not, may point to an increased IRIS susceptibility. In this case, the CNS-IRIS patient presented lower percentages of memory cells among CD4^+^ and among CD8^+^ T cells, and higher levels of activation of CD4^+^ T cells, at baseline. Contrary to our results, the retrospective study by Antonelli et al*.* [26] that compared 16 HIV-infected patients with different forms of IRIS with 29 HIV-infect patients without IRIS, showed no differences on baseline T cells populations. This inconsistency may be explained by the heterogeneity of IRIS patients included in that retrospective study (tuberculosis-IRIS, cryptococcosis-IRIS and others).

In general terms, the expected immune recovery after HAART initiation implies restoration of the CD4^+^ T cell count, which is characterized by two phases: 1) firstly, recirculation of activated memory T cells that had previously been sequestered in lymphoid tissues (cell redistribution); 2) secondly (4–6 weeks later or longer in patients with advanced disease), increase in the naïve CD4^+^ T cell counts related to improved thymopoiesis [27–29]. Accordingly, we observed an increase in the percentage of memory CD4^+^ T cells after 1 month of HAART, in both CNS-IRIS case and controls, but the former showed a higher increase than the latter. On a report from Antonelli et al*.* [26], no differences were observed on the absolute number of memory CD4^+^ T cells between IRIS and non-IRIS HIV-infected patients, though a higher proportion of effector memory cells among CD4^+^ T cells was reported in IRIS patients compared to patients that did not develop IRIS. Altogether, this data suggests that IRIS might be associated with an altered immune recovery, though it would be of much relevance to assess T cell function to further clarify if these cells underlie IRIS development.

One hallmark of IRIS pathogenesis, independently of the clinical presentation and associated pathogen, is an excessive activation of the immune system [23, 26]. We studied the expression of two surface activation-associated molecules on T cells: CD69, the earliest marker acquired after cell activation, and HLA-DR, a MHC class II molecule that is over-expressed later [30]. Our data support the hypothesis that higher activation of CD4^+^ T cells can be associated with a higher susceptibility to IRIS or be a feature of IRIS pathogenesis, but further studies are needed. Despite the activation profile of T cells on the CSF of the IRIS patient was not assessed, no major alterations would be expected as a normal cell count was observed. This data is in accordance with previous reports, where normal cell counts were observed on CSF of 2 patients with toxoplasmosis-associated CNS-IRIS not withstanding the presence of an intense inflammatory profile on the brain biopsies of those patients [31, 32].

Treg are known for their suppressive effect on the immune system [33]. The role of Treg in pathogenesis of HIV infection is very controversial. On one hand, Treg may have a beneficial role by downregulating immune activation and minimizing damage to self-tissues. Several studies showed that Treg correlate with decreased levels of T cell activation in HIV-infected patients [34–36], but at least one study showed a positive correlation between Treg proportion in blood and CD4^+^ T cell activation [37]. On the other hand, Treg may be detrimental by weakening the host immune response against HIV [38, 39] and serving as reservoirs for the virus [40]. However, there is also evidence that Treg directly inhibit HIV replication in activated T cells [41] and that protective HIV-specific CD8^+^ T cells evade Treg cell suppression [42]. Therefore, it seems that Treg play a dual role in the progression of HIV infection, as reviewed elsewhere [10, 43, 44]. There is some evidence that Treg present quantitative and qualitative alterations in IRIS cases [26, 45], but there is no consensus about their role in this condition. In our CNS-IRIS case, there was a decrease, followed by an increase, of the Treg percentage among CD4^+^ T cells. An increased proportion of Treg was previously observed in patients suffering from other types of IRIS, including mycobacterial- and cryptococcal-related IRIS [45, 46]. While an impaired Treg function could explain the excessive inflammation that characterizes IRIS, this has not been convincingly demonstrated. Unfortunately, no functional tests were performed to evaluate Treg suppressive activity in our CNS-IRIS case due to sample scarcity. Further studies on the evolution of Treg subsets and function in IRIS are required.

## Conclusion

Clinicians need to remain aware that HIV-infected patients with CNS toxoplasmosis are at risk of developing CNS-IRIS upon HAART initiation. Although this is a rare condition, it may lead to long-term sequelae and increased mortality, and this report may be useful for those facing the challenge of a potential CNS-IRIS diagnosis. In this clinical context, the use of MRI in association with clinical and laboratory data can reduce the number of unnecessary cerebral biopsies; additionally, corticosteroid therapy might not be necessary as the patient successfully recovered from his condition without it. Though we observe some alterations on the blood T cell subsets analysis that could underlie toxoplasmosis CNS-IRIS pathogenesis, the immunophenotypical characterization of other toxoplasmosis CNS-IRIS cases, as well as assessment of T cell function, will be essential to shed some light on the pathogenesis of this condition. A deeper knowledge on the mechanisms behind CNS-IRIS will help to define biomarkers for diagnosis and/or prognosis of this syndrome, helping clinicians to provide a more effective care.

## Additional file

Additional file 1: Supplementary Materials. (PDF 2892 kb)

## Abbreviations

AIDS: Acquired immunodeficiency syndrome
CNS: Central nervous system
CSF: Cerebrospinal fluid
CT: Computed tomography
HAART: Highly active antiretroviral therapy
HIV: Human immunodeficiency virus
IRIS: Immune reconstitution inflammatory syndrome
MRI: Magnetic resonance imaging
PBMCs: Peripheral blood mononuclear cells
PCR: Polymerase chain reaction
RTE: Recent thymic emigrants
Treg: Regulatory T cells
