# Supplementary material for: Toxoplasmosis-associated IRIS involving the CNS: a case report with longitudinal analysis of T cell subsets
Source: BMC Infect Dis. 2017 Jan 13;17:66. doi: 10.1186/s12879-016-2159-x (PMC5237164; doi:10.1186/s12879-016-2159-x)
Supplement: Supplementary file 1 — Supplementary Materials. (PDF 2892 kb) [file 12879_2016_2159_MOESM1_ESM.pdf]

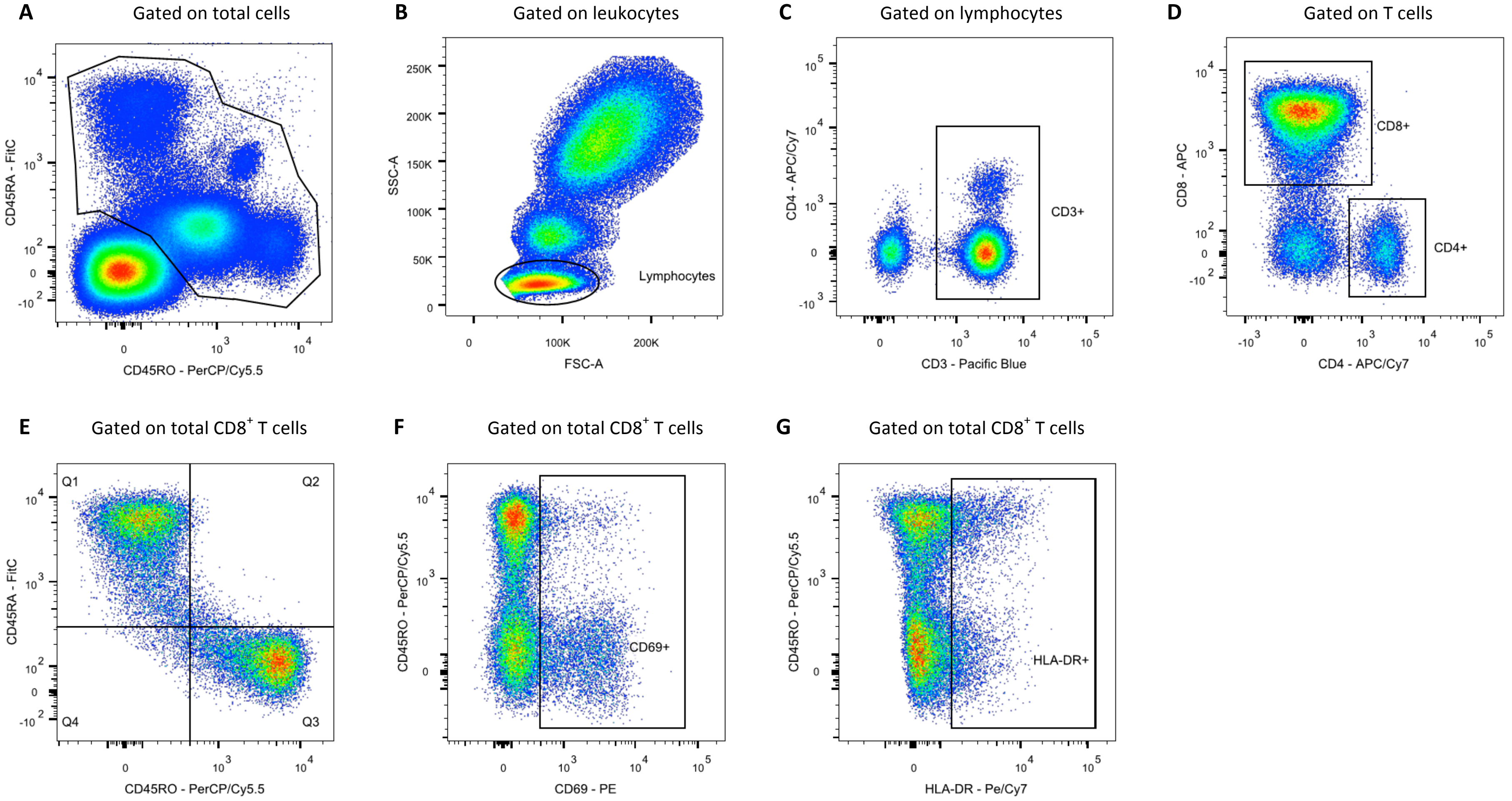

Figure S1. Flow cytometry gating strategy for analysis of T cell subsets. A representative example of the gating strategy used for the phenotypic characterization is shown. Cellular debris and non-leucocytes were excluded as CD45RA$^-$CD45RO$^-$ (**A**); among leukocytes (CD45RA$^+$ and/or CD45RO$^+$), lymphocytes were identified based on FSC/SSC profile (**B**). T cells (CD3$^+$) were selected from lymphocytes (**C**) and divided in CD4$^+$ and CD8$^+$ cells (**D**). Naïve and memory T cells were defined as CD45RA$^+$CD45RO$^-$ and CD45RA$^-$CD45RO$^+$, respectively, among CD8$^+$ T cells (**E**). Expression of the activation markers CD69 and HLA-DR was analyzed among total CD8$^+$ cells (**H** and **I**). Gates from naïve, memory, HLA-DR$^+$ and CD69$^+$ cells were transposed to CD4$^+$ T cells (not shown).

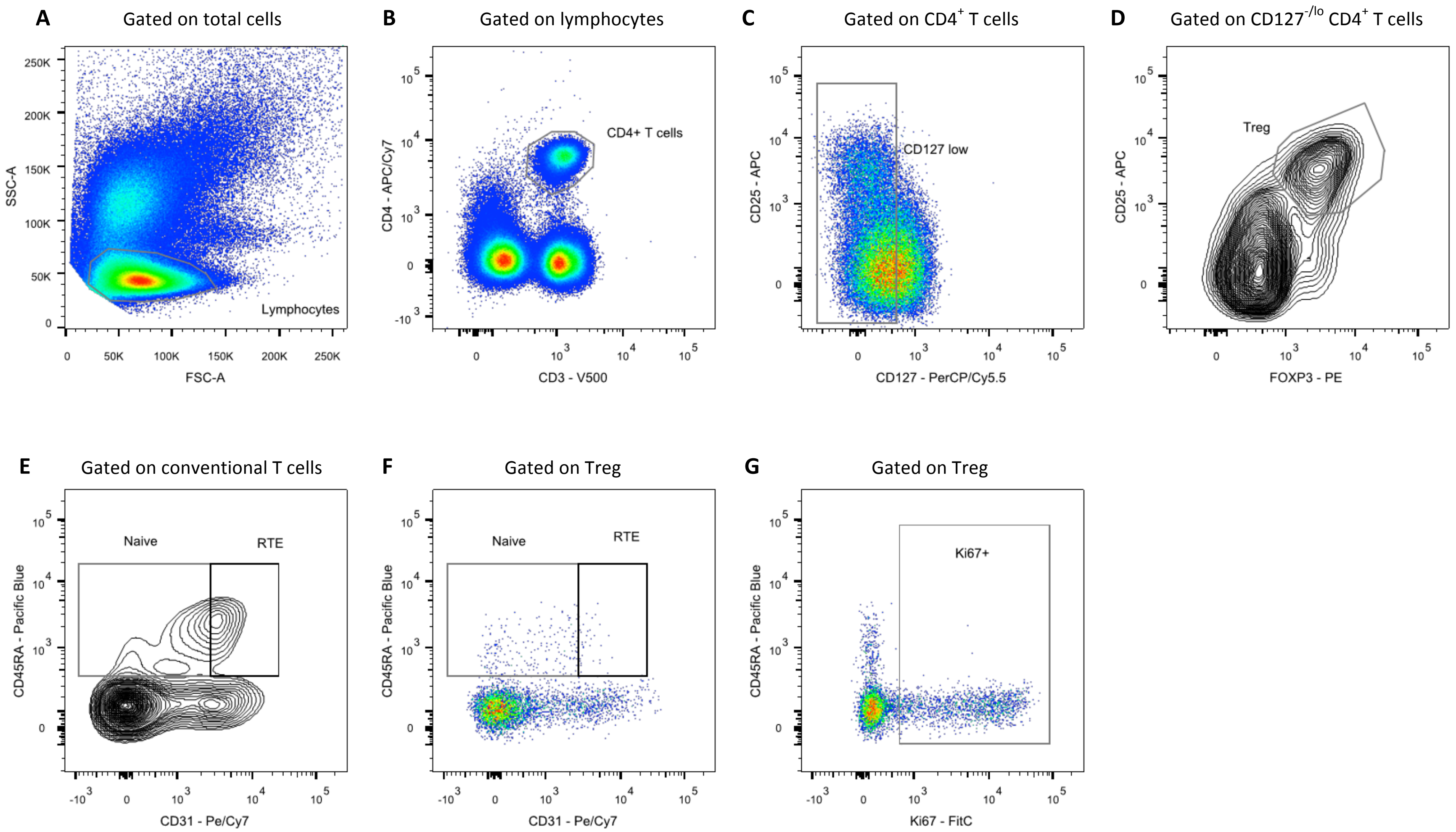

Figure S2. Flow cytometry gating strategy for analysis of regulatory T cells (Treg). A representative example of the gating strategy used for the phenotypic characterization of Treg is shown. Lymphocytes were identified based on FSC/SSC profile (**A**) and CD4^{+} T cells (CD4^{+}CD3^{+}) were selected within these (**B**). Treg were defined as CD127$^{-/low}$ CD4^{+} T cells (**C**) that were CD25^{+}FOXP3^{+} (**D**). Conventional T cells (Tconv) were defined using a "no gate" from Treg (not shown). Naïve (CD45RA^{+}) and recent thymic emigrant cells (CD45RA^{+} CD31^{Hi}) were selected within conventional T cells (**E**) and gates were transposed to Treg (**F**). Ki67 expression was analyzed within Treg (**G**).

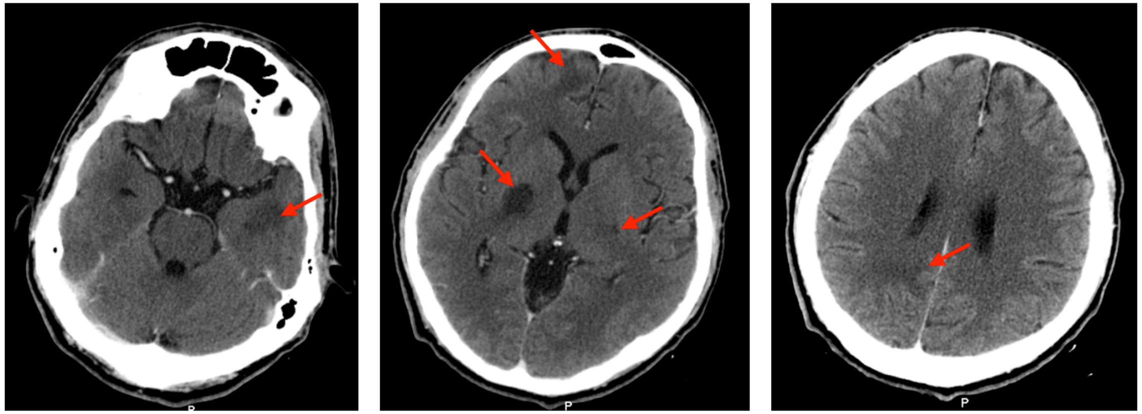

Figure S3. Brain CT performed after 14 days under specific anti-toxoplasma therapy, after contrast injection. Improvement of previous lesions is seen; mass effect and ring contrast enhancement are no longer evident.

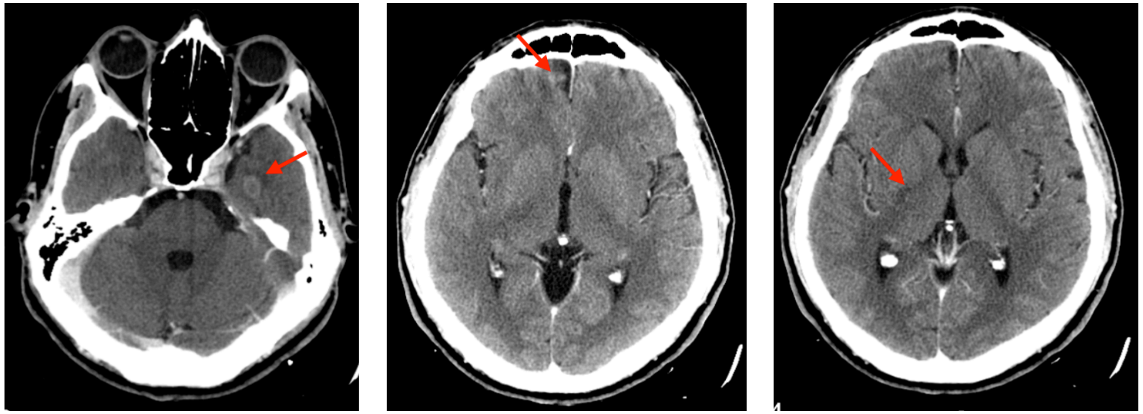

Figure S4. Brain CT performed after hospital admission, after contrast injection.
Several hypo-density lesions are present, some of them with slight perilesional edema and ring enhancement.

Table S1: Comparison between the T cell subsets dynamics of control group and the patient with toxoplasmosis associated CNS-IRIS.

			Baseline			1 Month			2 Months			3 Months			6 Months		
			Case	Controls[1]	*p* value[2]	Case	Controls[1]	*p* value[2]	Case	Controls[1]	*p* value[2]	Case	Controls[1]	*p* value[2]	Case	Controls[1]	*p* value[2]
Absolute count (cells/µL)	CD4$^+$	**Total CD4$^+$**	20.0	38.3 ± 30.7	*p*=0.111	67.0	109.0 ± 65.2	*p*=0.223	143.0	137.2 ± 58.9	*p*=0.776	128.0	107.4 ± 57.9	*p*=0.471	165.0	136.1 ± 44.6	*p*=0.088
		**Naïve**	3.2	3.4 ± 3.3	*p*=0.885	0.4	9.6 ± 10.6	*p*=0.122	1.0	7.1 ± 7.8	***p*=0.048**	3.2	8.2 ± 6.8	*p*=0.172	26.2	15.3 ± 11.7	***p*=0.024**
		**Memory**	14.0	32.5 ± 26.6	*p*=0.071	65.1	92.7 ± 53.9	*p*=0.316	137.6	124.4 ± 54.1	*p*=0.484	122.2	89.0 ± 47.9	*p*=0.196	129.4	112.9 ± 37.8	*p*=0.226
		**CD69$^+$**	7.1	7.4 ± 9.2	*p*=0.933	15.4	19.8 ± 22.2	*p*=0.679	5.0	12.6 ± 6.6	***p*=0.009**	23.2	15.5 ± 8.9	*p*=0.126	31.7	15.4 ± 8.1	***p*<0.001**
		**HLA-DR$^+$**	13.8	15.5 ± 15.5	*p*=0.748	38.3	45.9 ± 40.4	*p*=0.697	51.5	60.2 ± 29.9	*p*=0.408	79.5	43.4 ± 25.8	***p*=0.035**	53.1	40.8 ± 13.3	***p*=0.025**
	CD8$^+$	**Total CD8$^+$**	1186.0	746.6 ± 415.0	***p*=0.013**	1078.0	1288.8 ± 537.6	*p*=0.430	1308.0	1087.0 ± 448.7	*p*=0.178	852.0	920.4 ± 384.8	*p*=0.711	1132.0	957.6 ± 590.9	*p*=0.402
		**Naïve**	498.0	207.1 ± 155.2	***p*<0.001**	538.0	377.8 ± 203.8	*p*=0.154	695.0	329.8 ± 226.5	***p*=0.001**	344.0	297.6 ± 219.0	*p*=0.660	503.0	326.4 ± 256.1	*p*=0.104
		**Memory**	442.0	391.0 [191.0; 1446.0]	*p*=0.314	314.0	780.0 ± 646.7	*p*=0.182	419.0	458.0 [155.0; 1447.0]	*p*=0.214	244.0	313.0 [216.0 ; 977.0]	*p*=0.080	237.0	462.3 ± 296.3	*p*=0.052
		**CD69$^+$**	248.0	173.8 ± 103.4	*p*=0.063	232.0	266.2 ± 154.0	*p*=0.645	84.0	154.8 ± 64.0	***p*=0.011**	159.0	184.6 ± 118.0	*p*=0.653	191.0	138.1 ± 83.1	*p*=0.093
		**HLA-DR$^+$**	306.0	170.0 [28.9; 966.0]	*p*=0.110	344.0	210.0 [181.0; 837.0]	*p*=0.686	229.0	333.9 ± 220.5	*p*=0.191	264.0	195.0 [140.0; 666.0]	*p*=0.500	307.0	235.7 ± 193.3	*p*=0.300
Percentage (%)	CD4$^+$	**Naïve** (% among CD4$^+$)	15.9	9.9 ± 8.7	*p*=0.073	0.6	5.1 ± 5.4	***p*=0.050**	0.7	4.6 ± 4.8	***p*=0.041**	2.5	5.2 ± 4.1	*p*=0.083	15.9	10.5 ± 7.2	*p*=0.053
		**Memory** (% among CD4$^+$)	69.9	81.9 ± 10.9	***p*=0.011**	97.1	89.5 ± 7.8	***p*=0.029**	96.2	93.3 [75.5; 97.5]	***p*=0.015**	95.5	92.2 [71.3; 95.0]	***p*=0.008**	78.4	83.6 ± 10.0	*p*=0.155
		**CD69$^+$** (% among CD4$^+$)	35.7	16.0 ± 7.9	***p*<0.001**	23.0	10.0 [6.6; 38.3]	*p*=0.069	3.5	9.9 ± 5.2	***p*=0.006**	18.1	11.8 ± 5.1	***p*=0.006**	19.2	9.3 [7.2; 21.5]	***p*=0.021**
		**HLA-DR$^+$** (% among CD4$^+$)	69.0	36.8 ± 15.1	***p*<0.001**	57.2	42.2 ± 19.1	*p*=0.061	36.0	43.9 ± 10.1	***p*=0.046**	62.1	39.2 ± 14.5	***p*=0.001**	32.2	32.1 ± 10.9	*p*=0.986
	CD8$^+$	**Naïve** (% among CD8$^+$)	42.0	31.0 ± 18.3	*p*=0.107	49.9	32.5 ± 21.01	*p*=0.052	53.1	31.4 ± 19.9	***p*=0.011**	40.4	32.9 ± 20.3	*p*=0.300	44.4	34.1 ± 19.7	*p*=0.155
		**Memory** (% among CD8$^+$)	37.3	48.4 [39.2;85.3]	***p*=0.008**	29.1	57.4 ± 24.4	***p*=0.014**	32.0	55.2 ± 18.8	***p*=0.006**	28.6	50.5 ± 15.4	***p*=0.003**	20.9	50.0 ± 17.8	***p*=0.001**
		**CD69$^+$** (% among CD8$^+$)	20.9	23.7 ± 7.0	*p*=0.262	21.5	17.7 [11.5; 37.2]	*p*=0.208	6.4	15.0 ± 5.6	***p*=0.002**	18.7	15.8 ± 5.5	*p*=0.151	16.9	11.9 [10.9; 24.0]	*p*=0.173
		**HLA-DR$^+$** (% among CD8$^+$)	25.8	27.3 ± 14.1	*p*=0.752	31.9	30.6 ± 19.0	*p*=0.856	17.5	29.8 ± 10.7	***p*=0.009**	31.0	25.8 ± 8.8	*p*=0.116	27.1	22.0 [9.7; 50.3]	*p*=0.110

[1] Mean ± SD are represented for variables normally distributed; median and [min; max] are represented for the variables not normally distributed.

[2] For normally distributed variables, comparison of the control group with the clinical case was performed by one-sample T-test; for the other variables a one-sample Wilcoxon signed rank test was applied. Cells in bold show significant differences (p<0.05) between the value of the clinical case and the mean/median of the control group.

Table S2: Comparison between regulatory T cell (Treg) subsets dynamics of control group and the patient with toxoplasmosis associated CNS-IRIS.

		Baseline			1 Month			2 Months			3 Months			6 Months		
		Case	Controls[1]	*p* value[2]	Case	Controls[1]	*p* value[2]	Case	Controls[1]	*p* value[2]	Case	Controls[1]	*p* value[2]	Case	Controls[1]	*p* value[2]
Absolute count (cells/µL)	**Treg**	3.2	6.1 ± 5.1	*p*=0.155	4.9	12.4 ± 5.8	***p*=0.044**	17.4	23.7 ± 18.0	*p*=0.325	27.6	12.3 ± 6.8	***p*=0.007**	22.1	15.8 ± 7.5	***p*=0.036**
	**Naïve Treg**	1.1	0.1 ± 0.1	***p*<0.001**	0.2	0.5 ± 0.4	*p*=0.154	0.6	1.3 ± 1.0	*p*=0.086	3.8	0.9 ± 1.1	***p*=0.005**	3.23	1.5 ± 1.6	***p*=0.015**
	**RTE Treg**	0.8	0.0 ± 0.1	***p*<0.001**	0.2	0.2 ± 0.1	*p*=0.955	0.4	0.4 ± 0.3	*p*=0.824	1.0	0.3 ± 0.3	***p*=0.012**	0.8	0.4 ± 0.6	*p*=0.117
	**Ki67⁺ Treg**	0.9	1.3 ± 1.2	*p*=0.514	2.2	3.6 ± 2.2	*p*=0.230	5.51	4.8 ± 3.3	*p*=0.622	3.2	2.9 ± 1.9	*p*=0.781	5.66	2.8 ± 1.3	***p*<0.001**
Percentage (%)	**Treg** (% among $CD4^+$)	16.2	19.4 ± 9.4	*p*=0.362	7.4	18.9 ± 10.5	***p*=0.024**	12.2	16.4 [3.6; 38.7]	*p*=0.374	21.6	12.7 [8.9; 48.6]	*p*=0.110	13.4	12.3 ± 5.7	*p*=0.569
	**Naïve** (% among Treg)	33.6	1.7 ± 1.3	***p*<0.001**	4.3	3.8 ± 2.4	*p*=0.634	3.5	6.3 ± 5.8	*p*=0.214	13.6	2.5 [0.2; 15.8]	***p*=0.017**	14.6	5.4 [3.2; 25.0]	*p*=0.098
	**RTE** (% among Treg)	23.1	0.6 ± 0.6	***p*<0.001**	2.9	1.4 ± 0.8	***p*=0.001**	2.3	1.1 [0.6; 6.2]	*p*=0.161	3.5	1.1 [0.6; 5.2]	***p*=0.025**	3.5	1.7 [0.6; 11.0]	*p*=0.161
	**Ki67⁺** (% among Treg)	28.4	30.5 ± 19.4	*p*=0.820	44.8	28.3 ± 9.1	***p*=0.007**	31.6	25.2 ± 6.1	***p*=0.049**	11.6	20.0 ± 9.0	***p*=0.049**	25.6	15.3 [13.1; 35.9]	*p*=0.069

[1] Mean ± SD are represented for variables normally distributed; median and [min; max] are represented for the variables not normally distributed.

[2] For normally distributed variables, comparison of the control group with the clinical case was performed by one-sample T-test; for the other variables a one-sample Wilcoxon signed rank test was applied. Cells in bold show significant differences (p<0.05) between the value of the clinical case and the mean/median of the control group.
